# Paradigm Shifts in Surgery: Implications for Surgical Practice, Education, and Professional Identity

**DOI:** 10.1002/wjs.70266

**Published:** 2026-02-10

**Authors:** Hanoch Kashtan, Idan Carmeli, Yeal Feferman, Ran Orgad, Daniel Solomon

**Affiliations:** ^1^ Department of Surgery Assuta Ashdod University Hospital Ashdod Israel; ^2^ Faculty of Health Sciences Ben‐Gurion University of the Negev Be'er Sheva Israel

## 1 Introduction

Scientific and clinical disciplines evolve not only through incremental discoveries but also through fundamental shifts in how clinical problems are conceptualized and addressed. Thomas Kuhn's model of scientific revolutions, in which accumulating anomalies undermine a prevailing framework and give rise to a new paradigm, offers a useful lens through which to understand such transformations [1]. In surgery, where clinical decisions carry immediate and often irreversible consequences, paradigm shifts profoundly shape operative strategy, professional identity, and definitions of success. They also influence how surgeons are trained, how competence is defined, and how responsibility is shared within increasingly complex systems of care. Understanding prior paradigm shifts is therefore essential for preparing surgeons and trainees for future practice.

Here we examine selected paradigm shifts in surgical history and consider emerging forces that may catalyze the next conceptual evolution in surgical care.

## 2 Paradigm I: From Disrepute to Professional Authority

For much of Western history, surgery occupied a marginal and often stigmatized position within the healing professions. Surgeons were socially and professionally aligned with barbers and craftsmen, relying on empirical skills rather than scholarly training. This divide was reinforced by social hierarchies and academic prejudice that cast surgery as technical rather than intellectual.

This hierarchy began to erode through technical innovations and growing recognition of surgical achievement. A symbolic watershed occurred in 1686, when Charles‐François Félix successfully operated on Louis XIV of France for a perianal fistula, demonstrating that surgery could achieve outcomes beyond the reach of traditional medical therapies. In recognition, Félix was granted royal honors, and formal surgical teaching was authorized at Court, contributing to the establishment of the French Academy of Surgery.

By the 19th century, advances in anesthesia, antisepsis, and scientific methodology completed this transformation. Surgery became central to medical progress, and surgeons emerged as scientifically trained physicians. This paradigm shift laid the foundation for formal surgical training, academic credentialing, and the expectation that surgeons combine technical expertise with scientific reasoning.

## 3 Paradigm II: From Master Clinician to Multidisciplinary Collaborator

The early‐ and mid‐20th‐century surgeon embodied the “master clinician,” assuming responsibility for diagnosis, operative intervention, and postoperative management. This model reflected an era of bounded medical knowledge and modest specialization, in which professional autonomy was both expected and celebrated [2].

As medical knowledge expanded and specialization deepen, this paradigm became increasingly untenable. Complex diagnostics and advanced therapies exceeded the capacity of any single clinician. Early adaptations relied on consultation [3], but this proved insufficient. Multidisciplinary tumor boards and disease‐focused clinics emerged subsequently emerged [4], evolving into modern co‐management models in which surgeons partner with geriatricians, intensivists, and disease‐specific specialists [5].

This shift represents a genuine paradigm transformation: surgical care is no longer defined by the authority of an individual but by the structured integration of diverse expertise focused on patient outcomes. Contemporary surgical systems therefore require training surgeons not only as technical experts, but as effective collaborators within multidisciplinary teams.

## 4 Paradigm III: From Radical Extirpation to Biology‐Driven, Organ‐Preserving Cancer Surgery

The Halstedian paradigm dominated surgical oncology for nearly a century. Halsted proposed that cancer spread in an orderly centrifugal fashion, implying that increasingly extensive resections would improve outcomes [6]. Radical mastectomy became the archetype of this approach.

By the mid‐20th century, however, anomalies accumulated. Morbidity was substantial, while survival gains plateaued. Evidence increasingly suggested that micrometastatic disease often preceded surgery. Bernard Fisher and the NSABP trials initiated a scientific revolution by demonstrating equivalent survival between radical mastectomy and breast‐conserving surgery with radiotherapy [7], undermining the foundational logic of the Halstedian model.

This conceptual shift influenced multiple oncological disciplines, including colorectal, esophageal, and cervical surgery. Organ preservation, sentinel lymph node biopsy, neoadjuvant therapy, and multimodal treatment now exemplify a paradigm in which biological understanding rather than surgical radicalism, guides operative strategy. These changes have reduced morbidity while maintaining oncologic outcomes, reinforcing a shift toward value‐based surgical decision‐making and reshaping surgical education to emphasize biological literacy and interdisciplinary collaboration.

## 5 Paradigm IV: From “No Acid, No Ulcer” to the H. Pylori Revolution

For decades, peptic ulcer disease was conceptualized as an acid‐driven condition. The dogma “no acid, no ulcer” shaped both medical and surgical treatment, with vagotomy and related procedures constituting a major component of general surgery.

This paradigm unraveled as epidemiologic inconsistencies emerged. The discovery of H. pylori by Marshall and Warren contradicted assumptions of gastric sterility and demonstrated a bacterial etiology [8]. Antibiotic therapy rapidly replaced surgery, rendering most ulcer operations obsolete within a decade.

This paradigm shift illustrates how surgical practice can be transformed when disease conceptualization changes fundamentally. It underscores the need to train surgeons for adaptability and conceptual change, rather than mastery of static procedures.

## 6 Paradigm V: From Surgical Paternalism to Patient Autonomy and Shared Decision‐Making

Historically, surgical ethics were grounded in paternalism: the surgeon knew what was best. The technical complexity and urgency of surgery favored authoritative decision‐making, particularly when survival depended on rapid action.

The late 20th century rise of patient autonomy challenged this paradigm. Informed consent, biomedical ethics, and cultural shifts emphasized patient authority over bodily decisions [9]. As therapeutic options expanded, many surgical choices became preference‐sensitive, involving trade‐offs beyond technical expertise.

Shared decision‐making has thus emerged as a new paradigm, in which surgeons contribute clinical expertise while patients contribute values and priorities [10]. Surgical quality is now defined not only by technical success but also by alignment with what matters most to patients, requiring formal education in communication, ethics, and values‐based decision‐making alongside technical training.

## 7 Future Paradigms in Surgery

History cautions against confident predictions. Linear expectations have repeatedly collapse in the face of disruptive change. In 1894, The Times of London famously warned that the city streets would soon be buried under horse manure, failing to anticipate the automobile. Such episodes illustrate how linear expectations can be overturned by paradigm shifts, a caution that remains relevant as we consider the next era of surgical evolution. Examining past paradigm shifts may nevertheless reveal patterns that help anticipate future transformation.

Surgery now appears poised for profound change driven by technology, biology, and data science. Artificial intelligence may augment risk prediction, image interpretation, and intraoperative decision support. Virtual and augmented reality are reshaping surgical education through immersive simulation and procedural rehearsal. Precision oncology increasingly integrates tumor biology into decisions about operative timing and extent.

Advances in biofabrication and tissue engineering may alter reconstructive paradigms, while robotic and semi‐autonomous platforms suggest a shift from manual operation toward procedural oversight and integration.

Ethically, growing emphasis on patient autonomy and survivorship is redefining success. Traditional endpoints centered on survival are increasingly complemented by patient‐reported outcome measures, foregrounding function, symptoms, and quality of life.

Together, these developments suggest that future surgical paradigms will arise from the convergence of conceptual, technological, and ethical change.

For surgeons and educators, these forces argue for conceptual humility, interdisciplinary openness, and broader definitions of success beyond technical outcomes. Surgical education must therefore move beyond procedural proficiency toward cultivating adaptive expertise, ethical reasoning, and conceptual flexibility in the face of inevitable paradigm change.

## Author Contributions

**Hanoch Kashtan:** conceptualization, writing – original draft, writing – review and editing. **Idan Carmeli:** writing – review and editing. **Yeal Feferman:** writing – review and editing. **Ran Orgad:** writing – review and editing. **Daniel Solomon:** writing – original draft, writing – review and editing.

## Funding

The authors have nothing to report.

## Conflicts of Interest

The authors declare no conflicts of interest.

## Data Availability

Data sharing not applicable to this article as no datasets were generated or analyzed during the current study.
